# Recent highlights in the immunomodulatory aspects of Treg cell-derived extracellular vesicles: special emphasis on autoimmune diseases and transplantation

**DOI:** 10.1186/s13578-022-00808-4

**Published:** 2022-05-23

**Authors:** Yahya Asemani, Sajad Najafi, Fatemeh Ezzatifar, Naime Majidi Zolbanin, Reza Jafari

**Affiliations:** 1 Student Research Committee, Department of Immunology, School of Medicine, Shahid Beheshti University of Medical Sciences, Tehran, Iran; 2 Student Research Committee, Department of Medical Biotechnology, School of Advanced Technologies in Medicine, Shahid Beheshti University of Medical Sciences, Tehran, Iran; 3 Molecular and Cell Biology Research Center, Department of Immunology, School of Medicine, Mazandaran University of Medical Sciences, Sari, Iran; 4 Experimental and Applied Pharmaceutical Sciences Research Center, Urmia University of Medical Sciences, Urmia, Iran; 5 Department of Pharmacology and Toxicology, School of Pharmacy, Urmia University of Medical Sciences, Urmia, Iran; 6 Cellular and Molecular Research Center, Cellular and Molecular Medicine Institute, Urmia University of Medical Sciences, Urmia, Iran; 7 Hematology, Immune Cell Therapy, and Stem Cell Transplantation Research Center, Clinical Research Institute, Urmia University of Medical Sciences, Urmia, Iran

**Keywords:** Extracellular vesicles, Treg cells, Inflammation, Autoimmune, Transplantation

## Abstract

In order to maintain immunological tolerance to self and non-self antigens, one’s T regulatory (Treg) cells play a critical role in the regulation of detrimental inflammation. Treg cells inhibit the immune system in a variety of ways, some of which are contact-dependent and the others are soluble factors. Extracellular vesicles (EVs) are mainly secretory membrane structures that play a pivotal role in intercellular communication in both the local and systemic environments, enabling the transport of proteins, lipids, and nucleic acids between immune and non-immune cells. A number of studies have shown that Treg-derived EVs are specially formulated intercellular exchanging devices capable of regulating immunological responses by producing a cell-free tolerogenic milieu. Some of the processes suggested include miRNA-induced gene shutdown and upmodulation, surface protein activity, and enzyme transfer. Instead of being influenced by external circumstances like Tregs, exosomes’ cohesive structure allows them to transmit their charge intact across the blood–brain barrier and deliver it to the target cell with particular receptors. These properties have resulted in the use of Treg-derived EVs' immunomodulatory effects moving beyond laboratory research and into preclinical applications in animal models of a variety of inflammatory, autoimmune, and transplant rejection disorders. However, insufficient evidence has been produced to permit enrollment in human clinical studies. As such, we begin our research by introducing the most potent immunosuppressive elements discovered in Treg-derived EVs elucidating likely mechanisms of action in inhibiting immunological responses. Following that, we address recent research on the potential of suppressive EVs to regulate autoimmune inflammatory responses and improve tissue transplant survival.

## Background

The immune system's tasks include repelling infections and preventing the destruction of self-tissues (tolerance), as well as establishing the foundation for immunological responses. One of the most important regulatory subtypes involved in maintaining tolerance is CD4^Positive (Pos)^, CD25^Pos^, and Foxp3^Pos^ regulatory T cells (Tregs) [1]. More research on Tregs revealed their regulatory potential in virtually all immune processes, from infectious [2], oral [3] and fetal [4] tolerance to memory regulatory cell development [5]. Congenitally athymic nude mice with depleted Treg populations rejected allogeneic skin grafts [6], and the Foxp3 gene malfunctioned, causing fatal CD4^Pos^ T cell activation [7]. People with multiple sclerosis [8], IBD [9], atopic dermatitis [10], and insulin-dependent diabetes [11] have also been shown to have defective Tregs. By the way, a consensus of opinion among immunological researchers is that befitting communications between immune cells and other immune and non-immune cells are critical to inducing effective but yet meticulous responses in order to minimize undesirable subversions [12]. Along with cytokines and chemokines as the most imperative mediators involved in such immune communications [13], it is supposed that extracellular vesicles (EVs) also modulate the behavior of target cells [14, 15]. EVs are a heterogeneous family of mostly lipid-bound vesicles that differ in origin, size, shape, as well as content and are naturally released by nearly all living cells [16]. As multiple biologically active compounds constitute the main cargoes of EVs [17–19], they are progressively considered in several physiological as well as pathological processes [20]. So, it is thought that EVs, especially exosomes, may be able to help train the immune system [16, 21, 22]. Similar to other cell populations, immune cells also secrete EVs with specific deliveries that contribute to either switching off or on the other immune as well as non-immune cell activities [23]. T cells, as the conductors of almost all immune responses, also showed enhanced EV secretion following T cell receptor (TCR) engagement [24, 25].

Among the T cell population, Tregs, in addition to producing multiple immunosuppressive cytokines and metabolites, actively release EVs into the extracellular milieu that effectively suppress and modulate the activity of other innate and adaptive immune cells [26]. In a mouse model of kidney transplantation, it was shown that Treg-derived EVs repressed T cell proliferation dose-dependently and provided long-term allograft tolerance, especially following donor rather than recipient Treg-EVs administration [27]. Further research revealed that Treg-derived EVs are enriched sources of micro-RNAs that preferentially target Th1 cells, quelling their replication and IFNγ secretion in a mouse model of colitis [28]. Also, multiple sclerosis (MS) patients with recurrent attacks also showed dysfunctional Treg-derived exosomes that were unable to bridle autoreactive T cells [29, 30]. It can be inferred that the EVs actively secreted by Treg cells play a key role in modulating immune responses and participate in the process of multiple autoimmune and tolerance-based diseases. So, in the current paper, we try to unravel the immunomodulatory mechanisms of Treg-derived EVs/exosomes in the management of immune responses and address the dysregulated aspects of immunosuppressive EVs during autoimmune initiation and progress as well as transplant rejection. Finally, we offer some immunosuppressive EV-based therapeutic strategies to control and suppress autoimmune responses and avoid transplant rejection.

### Treg lymphocytes: subtypes and function in immunomodulation and tolerance

Tregs represent a ubiquitous group comprising both CD8^Pos^ and also CD4^Pos^ T lymphocytes with immunosuppressive capacities that modulate the innate as well as adaptive immune cell responses and bear the heavy burden of preserving self-tolerance. However, due to the lack of distinct differential markers from the other CD8^Pos^ T lymphocytes, most research has focused on the CD4^Pos^ subtype [31, 32]. Almost 5–10% of all CD4^Pos^ T cells have regulatory phenotypes [33] and the most common markers for Treg isolation and characterization are CD25 (IL-2 receptor chain) and the master regulator, Foxp3 [34–36]. Also, these cells are the tableau of other involved inhibitory molecules, including CD28, CD39, and CD73 (as ATPases), cytotoxic T lymphocyte antigen 4 (CTLA4), glucocorticoid-induced tumor necrosis factor-related receptor (GITR), and RANKL [33, 34]. Originally, Tregs were divided into two separate categories, including thymically (t) or naturally (n)-derived Tregs, as well as peripherally (p) or induced (i) Tregs [35]. As is clear, the first subset is mainly shaped under the influence of the moderate-to high-avidity encounter with self-antigens by T cell receptors (TCRs) and subsequent Foxp3 induction within the thymus [36]. Moreover, the recent thymic emigrants have the ability to act as preferential precursors of tTreg in the periphery [37]. The latter is developed from peripheral or in vitro antigen recognition by CD4^Pos^ T cells in both inflammatory and non-inflammatory conditions [38]. As well-known subtypes of Foxp3^Neg^ pTregs, T helper (h)3 cells and T regulatory type 1 (Tr1) impose their immunosuppressive potential primarily via the release of IL-10 but also TGF-β, respectively. Both Treg subsets harbor antigen specific TCRs, CD25, Foxp3, CTLA-4, and GITR and exhibit considerable immunosuppressive potential. The Helios transcriptional factor, neuropilin-1 (Nrp1), T cell immunoreceptor with Ig and ITIM domains (TIGIT), programmed cell death protein 1 (PD-1), CD73, as well as FcR-like 3 (FCRL3) are mainly applied to differentiate tTreg from the pTreg subset. However, Helios and Nrp1 are sometimes upregulated by pTregs under certain conditions [39, 40]. Also, Functional analysis of Treg cells has led to the identification of two distinct subpopulations, resting (CD62L^hight(hi)^CCR7^Pos^ or CD45RA^hi^CD25^low^) and effector (CD45RA^low^CD25^hi^ or CD62L^low^ CCR7^low^CD44^hi^KLRG1^Pos^CD103^Pos^) Tregs. The resting/naive or central Tregs are the most prominent circulatory and secondary lymphoid organ Treg cells with minimal immunosuppressive activities, while effector or activated Tregs with recent experience of antigen exposure show increased immunomodulatory abilities [41]. A newly discovered tissue-specific-resident Treg population with discrete functional and transcriptional plasticity from common lymphoid Tregs also has been shown to be necessary for tissue homeostasis and modulating local immune responses. This plasticity is highly dependent on the cytokine microenvironment as well as the type of encountering effector T cells that leads to optimal monitoring of the dynamics of the immune response in the involved tissue [42]. Another T cell subset with regulatory capabilities has recently been found, consisting of CD4^Pos^CD25^Negative (Neg)^ Foxp3^Neg^ HLA-G^Pos^ T cells. This cell subset exerts its suppressive actions primarily by soluble HLA-G production and higher levels of cytokine (both IL-10 and also IL-35) release [43]. As said, Tregs were initially discovered as decretive immune cells that were involved in preserving peripheral tolerance (limiting autoreactive T cells), nipping autoimmune reactions in the bud and extinguishing chronic inflammatory flares. However, their potential for limiting sterilizing immunity as well as anti-tumor responses has turned them into a double-edged sword [44]. Anyway, these beneficial and malefic activities are the result of several contact-dependent and independent mechanisms that can be easily categorized as: (1) immunosuppressive cytokines (IL-10, TGF-β, IL-35), (2) cytolysis and apoptosis of effector T cells (granzyme B and TNF-TNFR family members), (3) metabolic disruption of effector T cells (IL-2 depletion and adenosine generation) and (4) inducing tolerogenic dendritic cells (DCs). By the way, current research has presented novel immunoregulatory mechanisms via unveiling Treg as a rich source of EVs containing critical immunosuppressive biomolecules [26].

### EVs/exosomes in immune cell to cell communication

The EV term basically covers a mixed group of structures with disparate origins, size, content, shape, and biogenesis processes that most have a lipid bilayer membrane and are certainly secreted by all living cells. According to ascribed features like portaging cell specific receptors and carrying various noncoding RNA, miRNA, mRNA, DNA, proteins, lipids, and sugars, the EVs are considered as key mediators of intercellular communications and contribute to multiple physiological as well as pathologic events, including metabolic remodeling and tumor microenvironment adaptation, tissue regeneration and vascularization, and modulation of immune responses [45, 46]. Mostly, EVs are generated via outward budding of the cell membrane or by first inward budding of early endosomes and following the membrane fusion between the formed multivesicular bodies (MVBs) and the plasma membrane. Among EVs, MVs, apoptotic bodies, and exosomes have drawn the most attention in terms of research [26]. MVs with a diameter of about 100–1000 nm are generated by direct outward budding of the cell membrane and exert their effects through paracrine activities. Apoptotic bodies, ranging from 50 to 5000 nm, are separated from the dying cells, which leads to their earlier clearance by phagocyte scavenger receptors as well as modulation of inflammation. Exosomes, also known as intraluminal vesicles (ILVs), are formed by the inward budding of early endosomes that subsequently turn into MVB structures. The fusion of MVBs with the plasma membrane donates the release of these 30–150 nm exosomes to all body fluids that bear the ability of endocrine and systemic cell–cell communication. However, newer isolation and fractionation techniques have found new groups of exosomes, like small exosomes (60–80 nm), large exosomes (90–120 nm), and membrane-less exomeres (˂ 50 nm). Nevertheless, the potential of exomeres, particularly in immunological matters, remains unfamiliar [47].

Manifold immune cells also produce a lot of extracellular vesicles and particles (EVPs) that contain membrane and cytosolic elements. These extracellular vesicles and particles target a wide range of nearby and faraway recipient cells, and they play a big role in complex immunological processes like cell synapse, antigen presentation, T cell priming, metabolic rearrangement, and immunoregulation [48]. For more than two decades, the physiological and pathological roles of EVPs in innate and acquired immune responses have been exposed [49, 50]. A preliminary study of EVPs made by DCs showed that when they were released in an immune synapse, they made T cells more active [51]. Moreover, the EVPs' integrin and heat shock protein content greatly reduce tumor cell proliferation through T cell priming-dependent mechanisms [52]. During the MVB formation in antigen-presenting cells (APCs) like DCs or macrophages, the selective MHC-CD9 combinations are formed in their membrane and in the absence of APCs, the released EVPs can establish the same topology with stable and effective interactions with cognate T cells [53]. Also, the viral spreading and succedent uptake of such EVPs containing specific MHC-antigen complexes by other lymphoid DCs, provide their bystander sensitization and amplify CD4^Pos^ and CD8^Pos^ T cells promotion via selective TCR engagement [54]. However, the maturity stages of DCs can significantly affect the potential extent of T cell stimulation through related EVPs. The external surface of T cell-generated EVs are also rich in TCR/CD3 complexes and evoke the role of T cells for MHC I and II-bearing APCs [55]. Furthermore, the EVs encompass multiple integrins (such as LFA-1) and signaling pathway molecules (especially RAS/MAPK/ERK), and their crosstalk with various cell types confers specific consequences [56]. The EVPs from naive T cells mostly convey cytoskeletal compartments to target cells, while the activated T cells commonly secrete EVs with key metabolic as well as signaling components and transport them to mast cells and other resting T cells [57]. For example, the T cell-derived EV interaction with mast cells continues with MAPK pathway induction and subsequent release of IL-8 and oncostatin M [58]. Conversely, delivering the EVPs of T cells to DCs derives an immunosuppressive phenotype via downmodulating MHC I expression along with cell death via FAS/FASL contention [59]. B lymphocytes as professional APCs also secrete EVs that upmodulate the current T cell responses and, with their MHC class I load, collaborate with cross-priming DCs to induce CD8^Pos^ T cell responses [60, 61]. Interestingly, some B cell lymphomas bias the cells toward forming CD63^Pos^/MHC II^Pos^ EVs with IgG-antigen compacts that thwart common immunotherapies [62]. Immunoglobulins are the most frequent proteins in B cell-derived EVs and can be used to discriminate between healthy and cancer patients [63]. The extensive stimulatory and suppressive activities of immune cell-derived EVs and their multiple cellular targets are in arrears to their diverse content, including proteins, lipids, and nucleic acids [64]. Cytokines and chemokines, as the main protein components of immune EVs, are the critical mediators of immune cell–cell crosstalk and stimulation. The presence of either membranous or internally compacted cytokines within the EVPs not only enhances their lifespan by preventing enzymatic degradation, but also delivers them to a specific target cell [65, 66]. Among the nucleic acids in EVPs, the miRNAs are the most studied regulatory noncoding RNAs that can be shuttled between immune cells and modulate gene expression [67]. Albeit, the combination of wrapped miRNAs with specific mission and cell target differs between immune cells and almost depends on cell status. Sphingolipids, ceramides and less significantly cholesterol are major essential structural and non-structural lipids in immune EVPs. Evidence suggests that changes in the lipid arrangement of EVPs affect their attributed abilities [68, 69]. The immunosuppressive EVPs, as key mediators of diminishing immune responses as well as maintaining immune homeostasis and self-tolerance, are also secreted by several tumors, pathogens, and physiological stimuli [47]. In stable conditions, Treg-derived EVs are key players in adjusting self-tolerance and dampening chronic inflammatory responses [47]. Concerning this, research showed that EVs from Tregs promote tolerogenic DC induction and mitigate proliferation index and IFNγ secretion capacity of Th1 cells [28]. However, the activated CD8^Pos^ T cell-derived EVs with the FASL cargoes impose cytolysis of primed peripheral blood mononuclear cells (PBMCs) and suppress the activated DC responses [70].

### Treg cell-derived EVs/exosomes: contents and function in immunomodulation and tolerance

**Fig. 1:**
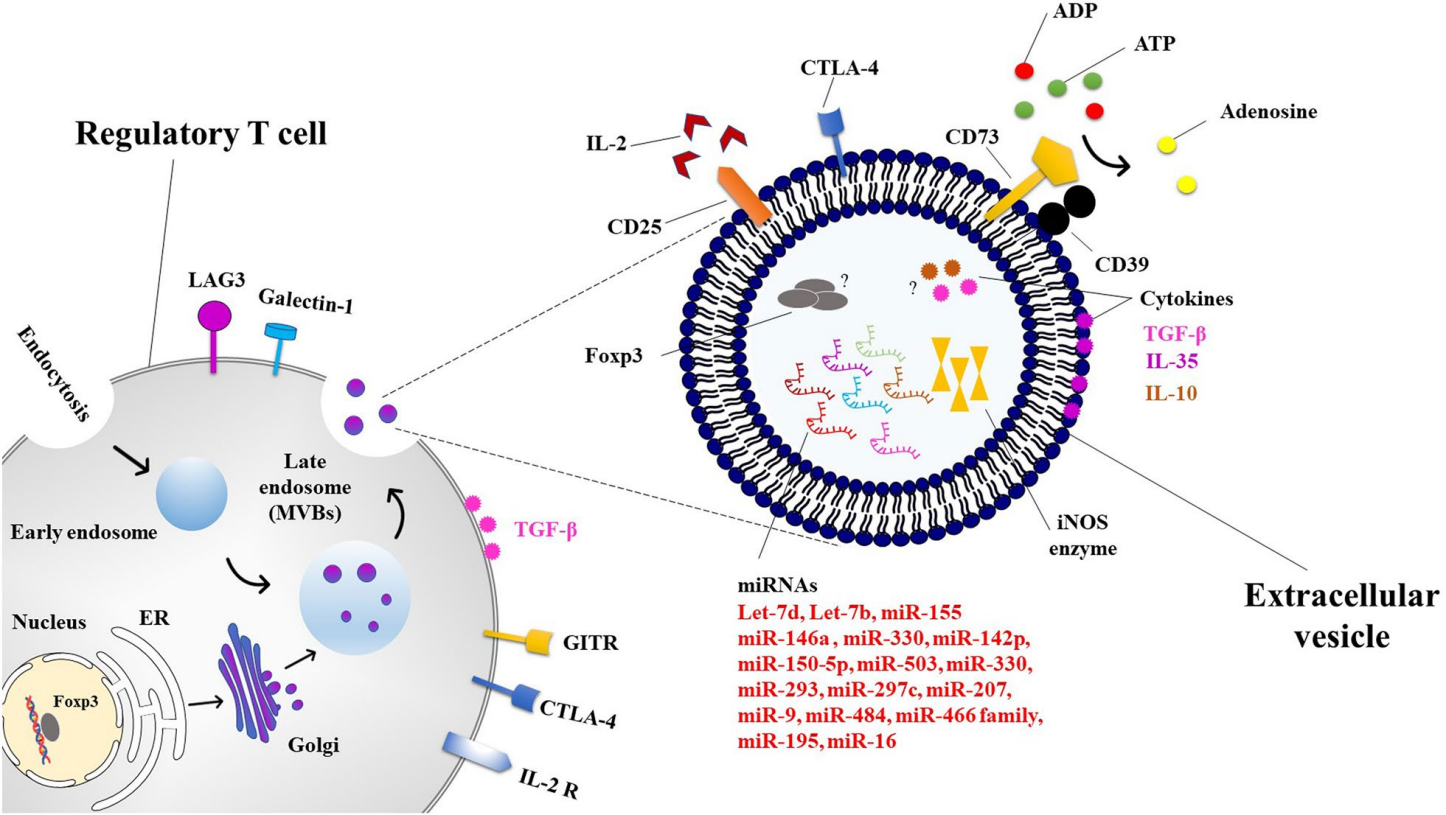
Investigated main components of Treg-derived extracellular vesicles with immunomodulatory and tolerogenic potentials. *ER* endoplasmic reticulum, *MVBs* multivesicular bodies, *LAG3* lymphocyte-activation gene 3, *GITR* glucocorticoid-induced TNFR family related gene, *CTLA-4* cytotoxic T-lymphocyte-associated protein 4, *IL-2R* interleukin 2 receptor, *ADP* adenosine diphosphate, *ATP* adenosine triphosphate, *iNOS* inducible nitric oxide synthase, *miRNA* microRNA or miR

Smyth et al. were the first to unravel the presence of immunomodulatory molecules such as CTLA-4, CD25, and CD73 in the Treg-derived EVs and acknowledged the need for CD73 in mediating the immunosuppressive activities of Tregs [71]. CTLA-4 is a negative regulator of the immunoglobulin superfamily that shows structural similarities to CD28 and constitutively expressed on the surface of Tregs [72, 73]. Compared to CD28, the extracellular binding domain of CTLA-4 binds to the B7-1 (CD80) and B7-2 (CD86) on the surface of APCs, albeit with much higher affinity [74, 75]. So, CTLA-4 is considered a critical regulator of T cell activity via antagonizing CD28 and competing in binding to cognate costimulatory ligands, recruiting phosphatases and other inhibitory signaling molecules to its cytoplasmic tail and collapse of the T cell-APC immunological synapse [76, 77]. Also, from in vitro result, it is supposed that the dampening propagation and activation of bystander T cells by Tregs is highly dependent on CTLA-4 [78]. CD25, through its sensible tendency, absorbs all the attainable IL-2 in the tissue microenvironment and not only sets the stage for Treg survival and proliferation, it also runs the mitochondrial apoptosis of other CD4^Pos^ T cells due to deprivation of the growth factor [79]. The CD73, as a membrane-bound ATPase, accelerates proinflammatory ATP to anti-inflammatory adenosine conversion, which inhibits cytokine release by T cells following interaction with the adenosine receptor [34]. Several studies have also shown that Treg-derived EVs transport miRNAs such as Let-7d, Let-7b, miR-155, and miR-146a-5p to target T cells and reduce their activation [28, 80]. Okoye group discovered that Foxp3^Pos^ Tregs can suppress Th1 cells by secreting let-7d-containing exosomes and that the upregulation of let-7d in Th1 cells inhibited their cell growth and IFNγ-secretion and aided in the control and protection of systemic diseases [28]. MiR-155 expression is significantly elevated within both T cells and B cells following antigen exposure and also APCs upon Toll-like receptor engagement, exhibiting its unique cell-detailed consequences upon transcriptional activation as well as cellular revenue [81–83]. It also modulates phenotypic dedication in CD4^Pos^ T cells and therefore plays a critical role in determining the combination of tolerance with immunity within humans [81, 84]. Particularly notable is that miR-155 is driven and induced via Foxp3 overexpression and is needed for proper thymic Treg formation [85]. Recent reports demonstrated that miR-155 maintains Treg cell competitive strength and conditioning by targeting SOCS1 toward expedited IL-2-facilitated activation of the transcription factor signal transducer and activator of transcription 5 (STAT5), although this is not required for proper regulatory T cells repressive actions [86]. MiR-155 is also expressed by conventional CD4^Pos^ T cells, and its expression levels affect susceptibility to tTreg suppressive activities [87]. MiR-155 expression has been found to be dysregulated in a variety of autoimmune and inflammatory disorders [92–94], as well as infertility problems associated with repeated implantation failure, fetal death, and preeclampsia, indicating that miR-155 may sway immunological adaptation for gestation [88–90]. As a result, the author postulated that miR-155 participates in effective maternal tolerance by altering Treg activity during fertilization. Besides, they showed in a separate study that miR-155 is necessary for prenatal Treg cell resource expansion in mice with genetic miR-155 loss, which has implications for Treg kinetics later in pregnancy. In mid-pregnancy, defective Tregs increase sensitivity to inflammation-induced fetal loss, which can be restored by injecting Treg cells of wild-type miR-155-sufficient animals. These results demonstrate that miR-155 is required for the progression of maternal Tregs during gestation and for the establishment of pregnancy tolerance [91]. The absence of miR-146a in Treg cells led to a collapse of immune tolerance, which manifested itself in a catastrophic IFN-dependent immune-mediated lesion in a number of tissues in the laboratory. Enhanced expression and activation of signal transducer and activator transcription 1 (STAT1), which is a direct target of miR146a, were most likely responsible for this increase. Additionally, enhanced Stat1 activation in Treg cells subjected to a SOCS1-specific deletion, a critical negative regulator of Stat1 phosphorylation downstream of the IFN receptor, was linked with analogues of Th1-mediated disease in a previous survey [92]. Without miR-146a, heart allograft life was prolonged and CD4^Pos^ and CD8^Pos^ T cell infiltration into the allografts was reduced. This had a modest tissue-protective impact. The lack of miR-146a, on the other hand, boosted Treg growth while impairing their capacity to limit T helper type 1 (Th1) responses. An interferon (IFN)-blocking and a miR-146a deficit worked together to restore Treg function, which in turn improved allograft survival and reduced rejection. Also, miR-146a controlled Tregs primarily via the IFN-/STAT1 pathway, which has been linked to Treg function in suppressing Th1 responses. MiR-146a regulation may improve Treg effectiveness in reducing heart transplant rejection in mice because of the findings that it regulates a particular component of Treg activity [93]. Additionally, previous research has shown that EVs produced from dominant negative (dn) IKK2-Treg cells contain miRNAs and the inducible nitric oxide synthase (iNOS) enzyme to target T cells and regulate their propagation and death. Indeed, they used dnIKK2-transfected DCs to develop a new population of tolerogenic CD4^Pos^CD25^Neg^ Treg cells that produce iNOS. MiR-503, miR-330, and miR-9 have been identified as the principal miRNA payloads of dnIKK2-Tregs that modulate cell cycle-regulating cyclins (E and D1) and T cell proliferation when NO is added. T cells exposed to EVs produced by dnIKK2-Treg cells also express Tim3 and release substantial quantities of IL-10. Additionally, EVs produced from dnIKK2-Treg cells inhibited the proliferation of IFN^Pos^ T cells, not only Th1 cells [101]. Tung's team revealed that EVs obtained from DC-induced Treg cells differed in their miRNA content analysis from control CD4^Pos^Foxp3^Neg^ T cells, with miR-150-5p and miR-142-3p being more often detected in EVs derived from DC-induced Treg cells. After in vitro stimulation of C57BL/6 Foxp3^Pos^Treg cells with allogeneic BALB/c DCs, the current study developed a murine Treg cell line that specifically targets BALB/c MHC class II molecule I-Ad antigens. In contrast to control Treg cells, the EVs of DC-induced Treg cells had no effect on the expression of the co-stimulatory molecule CD80 on DCs. The researchers observed that when DCs were stimulated with LPS, they produced less IL-6 and more IL-10, which was most likely due to miRNA transfer [94]. MiR-150 inhibits the development of T and B cells, as well as mature natural killer (NK) cells. When T cells are stimulated and differentiated into Type 1 (Th1) or Type 2 (Th2) T helper cells, miR150 levels drop. MiR-150 inhibits mRNAs involved in B cell development. Indeed, apoptosis is promoted by miR-150, which inhibits AKT3 and causes the buildup of the Bim signaling pathway. Reduced proliferation, increased apoptosis, and decreased activation of T cells are also shown in human CD4^Pos^ T cells with a high miR-150 level [95]. MiR-150 also targets c-Myb, which has diverse effects on NK and iNKT cells. A miR-150 transgenic with gain-of-function enhances the maturation of NK cells and increases their sensitivity to activation, reducing iNKT cells in the thymus and peripheral lymphoid organs [96]. W. Sang et al. found that miR-150 efficiently promotes immunological tolerance in allogeneic hematopoietic stem cell transplant patients by regulating CD4^Pos^ T cell activity. Thus, miR-150 levels may be used to predict the incidence of acute graft-versus-host disease [97]. Warth et al. observed that miR-99a represses mTOR, a recognized inhibitor of Treg cell development, but so does miR-150 through a cis-element. In vitro and in vivo, antagomirs decreased miR-150 expression and, therefore, Treg cell differentiation. Reconstrued cells lacking Dicer with miR-99a/150 indicated a novel mode of interaction. MiR-99a, which is highly expressed following retinoic acid activation of T cells, increases miR-150 target regulation. The mRNA 3′ UTR encodes this need for collaboration, which may be essential for miRNAs to influence cell fates even at low expression levels. So, it seems that miR-150 and miR-99a also work together to promote Treg induction by inhibiting mTOR signaling [98]. The miR-150-5p has previously been identified as a myasthenia gravis (MG) biomarker owing to its rise in serum and fall after thymectomy, which is associated with symptom improvement. Findings suggest that elevated miR-150 levels in MG patients may affect PBMC function. Additionally, anti-miR-150 treatment increased the expression of apoptotic genes within CD4^Pos^ and CD8^Pos^ T cells, demonstrating that miR-150 controlled their survival. Overall, miR-150 modulated target gene expression and peripheral cell survival, suggesting a function in MG at the thymic and peripheral levels [99]. Autophagy is one of the mechanisms via which miR-142-3p regulates natural Treg functionality [108]. Additionally, Gao et al. hypothesized that the aforementioned miRNA may act as an iTreg regulator. The expression of Foxp3, regulatory function, cytokine secretion, and death of in vitro activated T cells (iTregs) were all improved when miR-142-3p was reduced by antagomir. Suppression of miR-142-3p resulted in an increase in autophagy mediated by autophagy-related protein 16-1 (ATG16L1). The miR-142-3p target prediction and luciferase assay results revealed that miR-142-3p links unswervingly to lysine demethylase 6A (KDM6A), culminating in demethylation of H3K27me3 and increased production of the anti-apoptotic protein Bcl-2. As a result of these discoveries, the authors propose a novel strategy that includes silencing miR-142-3p in order to enhance the anti-apoptotic potential and function of iTregs by increasing KDM6A and Bcl-2 expression. This approach may be used to treat established persistent immune-mediated autoimmune and inflammatory diseases. In individuals with systemic lupus erythematosus, miR-142-3p has been shown to adversely influence T cell activation. ATG16L1 is upregulated in tTregs when miR-142-3p is knocked down, which leads to improved proliferation and immunosuppressive capabilities via targeting autophagy through overexpression of ATG16L1. Dekkema et al. also reported that higher expression of miR-142-3p in memory Tregs from patients with granulomatosis with polyangiitis (GPA) may be a contributing factor to their functional impairment via regulating ADCY9-mediated cAMP generation. According to the findings of this study, therapeutic interventions aimed at increasing miR-142-3p levels in Tregs while decreasing cAMP levels in Tregs represent a novel approach to restoring Treg function in GPA patients and, potentially, other autoimmune diseases in which there is a functional defect in the Treg subset [100]. Further research reveals that an axis of miR142-3p/Tet2/Foxp3 interferes with the successful induction of Treg cells during islet autoimmunity in models of type 1 diabetes, resulting in decreased Treg stability in both mice and humans. They have shown in non-obese diabetic mice that miR142-3p is increased in islet autoimmunity and that its restriction increases Treg induction and stability, resulting in decreased islet autoimmunity. Furthermore, utilizing a number of cellular and molecular approaches, they discovered that Tet2 is a direct target of miR142-3p and that high levels of miR142-3p are related to epigenetic remodeling in Tregs. These results provide a molecular hypothesis for autoimmune activation and development during islet autoimmunity, in which miR142-3p/Tet2-mediated Treg instability plays a role [101]. Cyclic AMP (cAMP) is a second messenger that is found throughout the body and controls a wide range of cellular processes. It has been discovered that CD4^Pos^CD25^Pos^ Tregs inhibit the activity of responder T cells by transferring cAMP to the cells in question. In study B. Huang et al. demonstrate that the miR-142-3p controls the synthesis of cAMP in CD4^Pos^CD25^Neg^ T cells and CD4^Pos^CD25^Pos^ Tregs by targeting the adenylyl cyclase (AC) 9 mRNA. MiR-142-3p limits the production of AC9 in CD4^Pos^CD25^Neg^ T cells, while Foxp3 regulates the level of cAMP in CD4^Pos^CD25^Pos^ Tregs by downregulating miR-142-3p, enabling the AC9/cAMP pathway to stay active. These results indicate a novel biochemical mechanism by which CD4^Pos^CD25^Pos^ Tregs maintain a high level of cAMP in order to perform their suppressor role, and they also show that the microRNA regulating AC expression may have an effect on the ultimate amount of cAMP in different cell types [102]. Additionally, it has been shown that the inability to manufacture effective EVs carrying anti-inflammatory cytokines and other inhibitory factors such as IL-35 and, most likely, IL-10, TGF-β and Foxp3 might contribute to the development and progression of a range of immune-mediated disorders. A current report by the Sullivan research team discovered that regardless of the expression of Foxp3 in IL-35-producing Tregs, they uniquely secreted EVs with decorated membranous IL-35 components (Ebi3 and p35), which promoted infectious tolerance in a novel way. To wit, the IL-35-coated EVs not only stimulated the same cytokine production in non-Treg cells, but they also imposed an immunosuppressive phenotype on T and B cells that have passively acquired surface IL-35, resulting in secondary suppression of immunological responses in the presence of the EVs [103]. Suppression of antigen-specific T cells by IL-10, a well-characterized suppressive cytokine of T cell proliferation and cytokine production, is required for peripheral tolerance to allergens, autoantigens, transplantation antigens, and tumor antigens [104]. Furthermore, this cytokine plays an important role in the development and maintenance of an anergic state, as shown by its ability to suppress graft-versus-host disease and allograft rejection in severe combined immunodeficiency patients who had human leucocyte antigen-mismatched bone marrow transplants [104]. The immunological tolerance to self-antigens in the CD4^Pos^ T cell subpopulation is maintained by the pleiotropic cytokine TGF-β, which has strong immunoregulatory effects. TGF-β-deficient mice acquire the phenotype of a fast-wasting illness leading to death at the age of 3 or 4 weeks due to activated CD4^Pos^ T lymphocytes in the blood. Even though it's debatable, TGF-β has been linked to the activation of Foxp3 in naive CD4^Pos^CD25^Neg^ T cells, converting them to CD4^Pos^CD25^Pos^ T cells. Evidence shows that TGF-β signaling is essential for CD4^Pos^CD25^Pos^ T cell in vivo growth and immunosuppressive capability. Researchers believe that TGF-β contributes to self-reactive clone death during lymphocyte maturation, as well as to tolerance maintenance, which both help to keep one from developing autoimmune disease [104] (Fig. 1). It should be noted, however, that while the inclusion of IL-10, Foxp3 and TGF-β in Treg exosomes has not yet been confirmed, the presence of the TGF-β on the surface of mesenchymal stem cell-derived EVs [105] as well as the inclusion of IL-10 and TGF-β in secreted exosomes by transduced DC cells with the mentioned cytokine genes [106, 107], suggest that Treg exosomes should most likely contain one or more of these factors.

### Treg cell-derived EVs/exosomes in translational medicine for immunomodulation of autoimmune diseases and transplantation

Tregs, as previously stated, contain immunomodulatory components and have the ability to adversely influence auto- as well as alloreactive responses. In 1990, it was firstly exposed by Hall et al. that elective transfusion of CD4^Pos^CD25^Pos^ Treg lymphocytes led to long cardiac allograft survival in treated rats with cyclosporine [108]. The mouse has been extensively used in immunotherapy research ever since, and more recently in preclinical human looking mouse models have been examined. As a result, numerous scientists engaged in future study attempted to uncover the potential of Treg EVs in animal models and preclinical humanized mice trials (Table 1). In an interrelated study by Yu et al., isolated donor and recipient Treg-derived exosomes were applied to evaluate their transplantation tolerance abilities in a kidney transplant rat model. The tolerogenic exosomes were extracted by sucrose/D2O density ultracentrifugation (110,000×*g*) and confirmed via transmission electron microscopy (TEM) analysis. The findings indicated that exosome release is a fundamental strategy of Treg immunosuppression and that donor exosomes have the biggest impact on inducing allograft tolerance. In vivo research, serum analysis, and histology revealed that the activity of donor exosomes may delay allograft rejection and extend the life duration of a transplanted kidney. Also, the exosomes were shown to have the ability to inhibit the growth of recipient T cells in in vitro examination. As a result, the research indicated that Treg exosomes may be used to decrease autoimmune reactions and improve transplant survival. However, the duration of immunological suppression against allografts caused by Treg exosomes remains unknown [27]. Another study by Okoye et al., demonstrated in a *Rag2*^−/−^ mice model of colitis and systemic inflammation that Treg-derived exosomes were required for suppression, but they were insufficient for effective immunomodulation by Tregs. The author's research group applied a combination of ExoQuick solution, ultracentrifugation, and serial centrifugation to separate the mentioned exosomes. Dynamic light scatter (DLS) was used to size the isolated exosomes, and the CD9, CD81, or CD63 antibodies were used in an ELISA to concentrate them. The provided EVs were preferentially uptaken by Th1 cells and affected their proliferation and cytokine release through miRNA transfer [28]. Similar research by Aiello et al., demonstrated that in vivo administration of dnIKK2-Treg-EVs improved kidney allotransplant survival by inducing a tolerogenic phenotype in conventional T cells and gravelling effector T cell development. After culturing the dnIKK2-Treg cells, they were able to purify the exosomes through several stages of centrifugation and then ultracentrifugation (100,000×*g*). The identity of the exosomes in this study was confirmed by western blotting with anti-CD63, -TSG101, and -calnexin antibodies as well as sorting flow cytometry and TEM analysis. So, it is possible that Treg-derived EVs will be used in the context of allograft transplantation as a tool for influencing the immune system and finding new immunosuppressive compounds. Apart from this, abnormalities in the synthesis of immunosuppressive EVs by Treg cells, as well as the release of defective exosomes, have been linked to the development of certain autoimmune disorders [109]. Based on prior research demonstrating insufficient regulation of autoreactive T cells due to impaired CD4^Pos^CD25^hi^ Treg cell function, Azimi et al., investigated whether exosomes produced by Treg cells in patients suffering from relapsing–remitting multiple sclerosis (RRMS), an autoimmune disease marked by demyelinating deterioration of the central nervous system, possessed diminished suppressive activity. They isolated exosomes from MS patients or control Treg cell supernatant and quantified them using a CD63 ELISA kit to determine their effect on the proliferation and survival of CD4^Pos^ T cells from RRMS patients. They observed that exosomes obtained from RRMS patients' Treg cells had less suppressive activity and triggered less death in conventional T cells than exosomes derived from healthy controls' Treg cells, suggesting for the first time that MS patient exosomes are malfunctioning (116). A humanized mouse skin transplant model demonstrated that human Treg-derived EVs protect tissue against alloimmune-mediated injury by limiting immune cell infiltration. A combination of serial centrifugation and ultracentrifugation, or ExoQuick-TC™ system, was employed for EV isolation. Subsequently, the isolated EVs were identified through TEM, NanoSight LM-10, and flow cytometry analysis. Further research showed that the secreted EVs also limited the aggressive proliferating T cells dose dependently and tipped the balance toward immunosuppressive IL-10 and IL-4 cytokines. As a whole, our findings suggest that Treg-derived EVs may be able to change the environment in a way that makes it easier for the body to fight off infections [79].

**Table 1:** Summary of Treg-derived EVs researches focusing on autoimmune diseases and transplantation models

Disease	Animal model/patient	EV source	EV content	In vitro outcome	In vivo outcome	Refs.
Kidney allograft	BN (RT1^n^) rat to Lewis (RT11) rat	MLN-derived Tregs	NS	↓ T cell proliferation	Lagging acute allograft rejection	[27]
Colitis and systemic Inflammation	Rag2^Neg/Neg^ mice	LO-derived Tregs	CD63, CD9, CD81 premature and mature miRNAs (including miR-466 family, miR-195, miR-16, Let-7d), T (h1) suppressive mRNA transcripts	↓ Th1 cell proliferation and IFNγ secretion	Suppression and prevention of systemic disease	[28]
Kidney allograft	BN (RT1n) rat to Lewis (RT11) rat	DnIKK2-Tregs	CD63, TSG101, iNOS, specific miRNAs (including miR-503, miR-330, miR-293, miR-297c, miR-207, miR-9 & miR-484)	↓ T cell proliferation, cell cycle arrest, apoptosis induction, naive to Treg cell conversion	Protracting kidney allograft survival	[109]
Relapsing–remitting multiple sclerosis	Human blood	PBMC-derived Tregs	CD63	Attenuated inhibition of conventional T cell proliferation and apoptosis	NS	[110]
Skin xenograft	Human to (Rag)2^Neg/Neg^γc^Neg/Neg^ (BRG) BALB/c mice	Human PBMC-derived Tregs	CD63, CD81, CD25, CD39, CCR4, CD4, CTLA-4, HSP90AB1, actin and tubulin, several miRNAs (including miR-142-3p & miR-150-5p)	↓ T cell proliferation, ↓ IFNγ, IL-2 and IL-6 secretion, ↑ IL-10 & IL-4 levels	Protracting skin xenograft survival	[79]

*EV* extracellular vesicle, *BN* Brown Norway, *MLN* mesenteric lymph node, *Rag2* recombination-activating gene 2, *LO* lymphoid organ, *miRNA* microRNA, *mRNA* messenger RNA, *Th* T helper, *DnIKK2* dominant-negative form of IKK2, *TSG101* tumor susceptibility gene 101, *iNOS* inducible nitric oxide synthase, *PBMC* peripheral blood mononuclear cell, *CTLA-4* cytotoxic T lymphocyte-associated antigen-4, *HSP* heat shock protein, *IFNγ* interferon gamma ↓: decrease, ↑: increase, *NS* not specified

## Conclusion

From the evidence collected here, it is obvious that EVPs produced by regulatory T cells retain the immunomodulatory properties of their respective parent cells. In addition, research has shown that these effects are caused by the presence of a number of ever-expanding surface and internal factors libraries with immunosuppressive characteristics, including IL-35, CTLA-4, CD73, miR-150, miR-146p, etc. The key question, however, is "will EVs generated by Treg cells be of particular interest to researchers in an immunotherapeutic clinical setting?" Before delving into the issue, as Kelly et al. contend, it is necessary to keep in mind that although Treg-derived EVs indeed mediate a substantial part of regulatory T cells' repressive capacity, these EVs alone cannot compensate. To exert maximal impact, the existence of additional contact-dependent and independent suppressive mechanisms is needed. Additionally, the milieu in which regulatory T cells reside and the kind of antigen that crosslinks their TCRs have an effect on the type and quantity of cytokines, miRNAs, and other inhibitory factors such as iNOS in their released EVPs. To put it another way, the immunotherapeutic potential of Treg-derived EVs as an alternative or supplementary treatment in situations when immunological tolerance restoration and/or readjustment is needed is not implausible. However, depending on the regulatory T cell type, the manner of purification and stimulation, and the cytokine environment, a varied population of EVPs will be generated, with each group displaying the proper functional state and therefore performing a range of specific roles that must be considered in all experimental settings. So far, the overwhelming majority of research that has progressed to the early phases of clinical trials has modulated immunity in patients using autologous amplified regulatory T cells or in vitro induced Tregs. The bottom line is that regulatory T cells, owing to their plasticity, may lose their identity and transform into invasive effector cells in response to the inflammatory milieu in patients' bodies. On the other hand, the quantity and composition of EVs generated may be altered, and regulatory T cells may be unable to reach the inflammatory nucleus. In return, EVs offer a number of benefits over cell-based treatment. For instance, EVs are more durable over time and, once injected in vivo, retain their phenotype, which is particularly advantageous in the inhospitable inflammatory tissue microenvironment. Moreover, since the EVs are safe to administer, can be tailored to target a particular cell type via specific receptors, and can also contain disease-modifying payloads, they hold great promise for the development of EV-based therapies in the future. Studies published to date have shown individual gifts results in increasing allotransplant survival and modulating inflammatory responses in animal models by regulatory EVs and exosomes. However, there is insufficient data to support their usage in clinical studies. The issue is that, for example, in investigations of allograft survival in the heart, skin, and kidney transplantations, researchers have utilized heterogeneous models of mice, rats, etc. as well as Tregs derived from various sources to decrease transplant rejection reactions. On the other hand, the use of separate EVP separation methods, as well as the employment of different markers to identify them, makes it somewhat more difficult to combine the findings and draw general conclusions. Because, at the moment, the application of fractionation methods and a range of ultracentrifugation gravities and durations has revealed subfamilies of EVs with diverse biological features, each of which will undoubtedly have its own implications. On the other hand, as shown in patients with multiple sclerosis, the creation of defective regulatory Treg-derived EVs is a fundamental component of inflammatory autoimmune disorders, which significantly expands our imagination about their potential as a modifiable therapeutic approach. On the whole, the findings of published preclinical studies indicate that Treg EVs are efficient at regulating inflammatory responses in autoimmune disorders, as well as decreasing alloresponses after transplantation and improving allograft survival. Additionally, the presence of inhibitory components, particularly microRNAs, in EVs supports this. However, the current data is insufficient for further clinical research in transplantation alongside inflammatory and autoimmune disorders. Additionally, standardizing and clarifying EVP separation methods, as well as the usage of Tregs derived from common sources, makes it simpler to assess their efficacy. Therefore, future research should include various models of tissue transplantation and autoimmune disorders in addition to the utilization of identified EVPs and comparable separation procedures.

## Data Availability

Not applicable.
